# Evolving paradigms in lymph node surgery in endometrial cancer: insights from the multicenter E-PEC trial

**DOI:** 10.1007/s00404-026-08514-z

**Published:** 2026-07-28

**Authors:** B. Eser, D. Papior, J. Salmanton-García, O. A. Cornely, B. Morgenstern, C. Herpel, J. C. Radosa, A. Almuheimid, B. Aktas, L. Weydandt, J. Wittenborn, P. Meyer-Wilmes, V. Friebe, C. Leidinger, P. Buderath, R. Kimmig, F. Thangarajah

**Affiliations:** 1 Department of Gynecology and Obstetrics, University Hospital of Essen, Medical Faculty of the University of Duisburg-Essen, Essen, Germany; 2 Institute of Translational Research, Cologne Excellence Cluster On Cellular Stress Responses in Aging-Associated Diseases (CECAD), Faculty of Medicine, University Hospital Cologne, University of Cologne, Cologne, Germany; 3 Clinical Trials Centre Cologne (ZKS Köln), Faculty of Medicine and University Hospital Cologne, University of Cologne, Cologne, Germany; 4 Department I of Internal Medicine, Center for Integrated Oncology Aachen Bonn Cologne Duesseldorf (CIO ABCD), University of Cologne, Cologne, Germany; 5 Department I of Internal Medicine, European Confederation for Medical Mycology (ECMM) Excellence Center, University of Cologne, Cologne, Germany; 6 German Centre for Infection Research (DZIF), Partner Site Bonn-Cologne, Cologne, Germany; 7 University of Cologne, Faculty of Medicine and University Hospital Cologne, Department of Gynecology and Gynecologic Oncology;, Center for Integrated Oncology (CIO Aachen, Bonn, Cologne, Düsseldorf), Bonn, CologneCologne, Düsseldorf Germany; 8 Department of Gynecology and Obstetrics, University Hospital of Homburg, Homburg, Germany; 9 Department of Gynecology, University Hospital of Leipzig, Leipzig, Germany; 10 Comprehensive Cancer Center Central Germany, Partner Site Leipzig, 04103 Leipzig, Germany; 11 Department of Obstetrics and Gynecology, Center for Integrated Oncology (CIO Aachen, Bonn, Cologne, Düsseldorf), University Hospital of RWTH Aachen, Aachen, Germany; 12 Department of Gynecology and Obstetrics, University Hospital of Düsseldorf, Düsseldorf, Germany

**Keywords:** Endometrial cancer, Sentinel-node biopsy, Surgical treatment, Lymph node assessment, Surgical trends

## Abstract

**Objective:**

To describe temporal trends in lymph node staging for endometrial cancer in selected German academic centers between 2018 and 2022.

**Methods:**

In this multicenter retrospective cohort study, patients who underwent primary surgical treatment for newly diagnosed endometrial cancer between 2018 and 2022 at six German university hospitals were included. Patients receiving primary systemic or chemoradiation therapy were excluded. Patient characteristics and treatment details were analyzed descriptively and stratified by year of surgery. This multicenter retrospective cohort study was conducted as part of the E-PEC project, an ethics-approved multicenter project assessing surgical management patterns in newly diagnosed endometrial cancer.

**Results:**

A total of 644 patients met the inclusion criteria. Overall, 62.4% underwent lymph node surgery. Sentinel-based procedures were increasingly performed over time and accounted for 61.4% of all nodal staging procedures. In contrast, the rate of primary systematic lymphadenectomy significantly decreased from 30.3% in 2018 to 17.9% in 2022 (*p* < 0.05).

**Conclusions:**

We observed a temporal increase in less radical lymph node procedures in selected German academic centers during the study period. Sentinel-based techniques were increasingly used over time in parallel with evolving guideline recommendations.

## What does this study add to the clinical work

**Table:** 

This multicenter retrospective E-PEC analysis demonstrates a shift in lymph node management for endometrial cancer between 2018 and 2022, with increasing use of sentinel-based procedures and decreasing primary systematic lymphadenectomy in German academic centers. These findings support the ongoing transition toward less invasive nodal staging in routine clinical practice, while emphasizing that institutional practice patterns and missing nodal data should be considered when interpreting surgical trends.	

## Background

Endometrial cancer is the most common gynecologic malignancy in developed countries and the fifth most common cancer in women worldwide. In Germany, an estimated 11,200 cases were predicted for 2020 [1]. About 75% were diagnosed at FIGO stage I, resulting in a relatively good prognosis with a 5 year survival rate of 78% overall [2]. Although lymph node metastases are uncommon in low-risk early-stage disease, nodal involvement may occur even in apparently early-stage tumors depending on histologic and molecular risk factors. Surgical lymph node staging is crucial for determining adjuvant therapy [3]. Over the past decade, treatment strategies have shifted toward less invasive approaches, including minimally invasive surgery and sentinel lymph node biopsy (SLNB), to reduce morbidity [4–11]. Sentinel-node mapping was first described over 20 years ago [8], and prospective trials have shown high diagnostic accuracy even in high-risk cases [9, 10].

Although incorporated into European Society of Gynaecological Oncology (ESGO) guidelines in 2021 and into German S3 Guidelines in 2022, respectively, data describing temporal changes in clinical practice before formal recommendations remain limited.

The number of ongoing studies comparing relapse and survival after sentinel lymphadenectomy versus systematic lymphadenectomy remains small. The E-PEC project ("Early implementation of practice-changing study results and guidelines in academic centers in the surgical treatment of patients with endometrial cancer") is a multicenter retrospective observational database established by the GO WEST study group. It includes patients undergoing primary surgical treatment for endometrial cancer between 2018 and 2022 at six German university hospitals. The overall objective of E-PEC is to describe temporal changes in surgical management and the implementation of emerging evidence and guideline recommendations in routine clinical practice.

## Patients and methods

This multicenter retrospective analysis was conducted within the E-PEC project and included patients who underwent surgery for newly diagnosed endometrial cancer between 2018 and 2022 at six German university hospitals. Patients who received primary chemoradiation or primary systemic therapy were excluded. All patients fulfilling the E-PEC eligibility criteria were screened for inclusion. The present analysis included all E-PEC patients undergoing primary surgery for whom data regarding lymph node management were available. The exclusion criteria applied were identical to those used within the E-PEC project.

Patient characteristics, including general demographic data, histological parameters, and treatment details, were systematically collected in the E-PEC database across all participating centers. Data focusing on lymph node assessment were extracted from the E-PEC database and subsequently analyzed using SPSS version 28.0 (IBM Corp., Armonk, NY, USA).

The primary focus of this analysis was to examine the evolving trends in the surgery of staging lymph nodes among patients with endometrial cancer. Specifically, the analysis focused on assessing temporal changes in surgical practices related to lymphadenectomy between 2018 and 2022. The objective of this analysis was to describe temporal trends in lymph node surgery between 2018 and 2022. The frequency and type of lymph node procedures were assessed descriptively across the study period.

Additionally, demographic and clinical characteristics of the patient cohort, such as age, body mass index (BMI), and tumor stage, were collected and analyzed to provide a comprehensive overview of the patient population included in the E-PEC database. Tumor staging was based on the original FIGO classification applicable at the time of treatment together with TNM staging (UICC/AJCC 8th edition). References to FIGO 2023 in the Discussion serve solely to contextualize the findings within current standards; no retrospective reclassification was performed [12].

The following types of lymph node surgery were performed:

- Primary systematic lymphadenectomy: this procedure entails the en bloc removal of all pelvic and paraaortic lymph nodes during the initial surgical intervention. Its primary objective is to provide comprehensive staging and facilitate accurate assessment of nodal metastasis [11].
- Secondary systematic lymphadenectomy: for the purposes of this analysis, secondary systematic lymphadenectomy was defined as systematic pelvic and/or paraaortic lymphadenectomy performed within the primary treatment pathway of newly diagnosed endometrial cancer after either an initially sentinel-based approach with suspicious or positive sentinel-node findings, or after initial omission of nodal staging when no indication for systematic lymphadenectomy was apparent at primary surgery, followed by secondary nodal staging after reassessment of the primary tumor risk profile. This category does not include procedures performed for recurrent or persistent disease [11].
- Sentinel-guided lymphadenectomy: this technique utilizes the injection of tracers to identify the sentinel lymph node(s) during systematic lymphadenectomy [5–7].
- Sentinel-node biopsy: the procedure involves the surgical removal of the sentinel lymph node(s), followed by detailed histopathological analysis to detect metastatic deposits. This minimally invasive approach aids in staging while reducing morbidity associated with extensive lymphadenectomy [5–7].
- Targeted compartmental lymphadenectomy: this method focuses on the selective sentinel-based dissection of lymph nodes and their afferent lymph vessels within specific anatomical compartments based on tumor location and known patterns of spread. It aims to optimize staging accuracy while minimizing unnecessary tissue removal and associated morbidity [5–7].

All procedures included in this analysis were performed in the context of primary treatment for newly diagnosed endometrial cancer.

For the purpose of analysis, categories of lymph node procedures were defined as mutually exclusive at the patient level. Patients in whom a nodal procedure was attempted or documented but no evaluable lymph node tissue was retrieved or confirmed on pathological examination were classified as Nx for TNM nodal staging and reported separately in Fig. 2. In cases where combined approaches were performed, patients were assigned to a single category based on the most extensive lymph node procedure.

Continuous variables are presented as mean ± standard deviation (SD) or median (SD), as appropriate. Categorical variables are reported as counts and percentages.

Comparisons of categorical variables across years were performed using chi-square tests or Fisher’s exact tests, as appropriate.

All statistical analyses were exploratory in nature. A p-value < 0.05 was considered statistically significant. No adjustments for multiple comparisons were performed.

Given the multicenter design, no adjustment for center-specific effects or patient-level confounders was applied.

Detailed information on sentinel lymph node mapping techniques, including tracer type (e.g., indocyanine green or blue dye), injection method, detection rates, laterality (unilateral vs. bilateral detection), and paraaortic mapping, was not consistently available across all participating centers. Therefore, these parameters could not be analyzed in a standardized manner within the present study.

In accordance with the journal’s guidelines, we will provide our data for independent analysis by a selected team by the Editorial Team for the purposes of additional data analysis or for the reproducibility of this study in other centers if such is requested.

The study “Early implementation of practice-changing study results and guidelines in academic centers in the surgical treatment of patients with endometrial cancer (E-PEC Trial)” was reviewed and approved by the Ethics Committee of the Medical Faculty, University of Duisburg-Essen, Germany (approval number 24–11932-BO, approved on July 2, 2024).

The E-PEC project was not prospectively registered in a public trial registry, as it represents a retrospective observational database study based on anonymized routine clinical data and not a prospective interventional trial.

The study involved only retrospective analysis of anonymized data from medical records, questionnaires, and routine imaging, in accordance with the Declaration of Helsinki.

## Results

In total, 644 patients met the inclusion criteria. Patient characteristics are summarized in Table 1. The median age was 63 years (standard deviation (SD):12.2) with a median BMI of 30 kg/m^2^ (SD:11.0).

**Table 1:** Patient characteristics

	Median	Standard deviation (SD)
Age (years)	63	12.2
BMI (kg/m^2^)	30	11.0
	n	Percentage (%)
TNM		
T-stage		
T1a	332	51.6
T1b	184	28.6
T2	59	9.2
T3a	30	4.7
T3b	35	5.4
T4	3	0.5
Unknown	1	0.2
N-stage		
N0	326	50.6
N1	58	9.0
N2	18	2.8
Nx	242	37.6
M-stage		
M0	546	84.8
M1	14	2.2
Mx	84	13.0
Grading		
G1	272	42.2
G2	195	30.3
G3	172	26.7
Gx	5	0.8
ER positive	338	52.5
PR positive	317	49.2

*BMI* body mass index; *TNM* tumor, node, metastasis; *ER* estrogen receptor; *PR* progesterone receptor

At diagnosis, the majority of patients (*n* = 516; 80.1%) were classified as FIGO stage I, representing the largest subgroup, followed by stage III with 71 patients (11.1%).

Tumor staging according to the Tumor, Node, Metastasis (TNM) classification revealed that most tumors were T1a (< 50% myometrial invasion) accounting for 332 cases (51.6%).

The second most common T-stage was T1b (> 50% myometrial invasion) with 184 cases (28.6%). Higher T-stages were less frequent; only three patients (0.6%) had T4 disease.

Regarding nodal involvement, the majority of patients (*n* = 326; 50.6%) had no lymph node metastases (N0). Pelvic lymph node involvement (N1) was observed in 58 patients (9.0%), and paraaortic lymph node involvement (N2) in 18 patients (2.8%). In 242 cases (37.6%), nodal status was classified as Nx. This included 238 patients who did not undergo lymph node surgery and four patients in whom a nodal procedure was attempted or documented intraoperatively, but no evaluable lymph node tissue was retrieved or confirmed on pathological examination. Consequently, these four patients could not be assigned a pathological nodal status and were classified as Nx. This distinction between surgical documentation and pathological nodal assessment should be considered when interpreting trends in nodal staging.

Distant metastases at diagnosis were absent in most patients: 546 patients (84.8%) had no evidence of metastasis (M0). Distant metastases were ultimately documented in 14 patients (2.2%) during final histopathological evaluation and/or postoperative diagnostic work-up. These patients were not known to have M1 disease prior to surgery. All underwent primary surgical treatment under the clinical assumption of localized disease and without preoperative evidence of distant metastasis. They were therefore retained in the analysis because their surgical management reflected primary treatment and staging of newly diagnosed endometrial cancer.

Tumor grading showed that G1 tumors were most prevalent, comprising 42.2% of cases (*n* = 272), followed by G3 tumors in 172 cases (26.7%). In five cases (0.8%), grading information was missing. The analysis of hormone receptor status revealed that the estrogen receptor was positive in 338 cases (52.5%), while progesterone receptor expression was observed in 317 cases (49.2%; see Table 1).

**Fig. 1:**
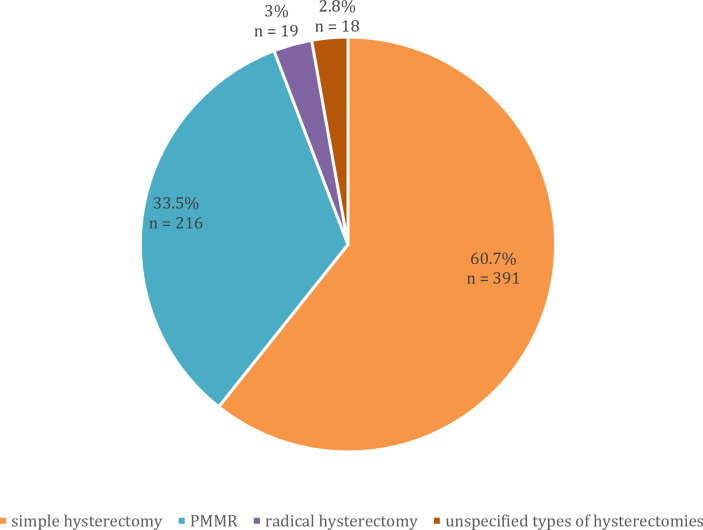
Distribution of different hysterectomy types

The hysterectomy was most commonly performed as a simple hysterectomy, accounting for 60.7% (*n* = 391) of the cases, followed by peritoneal mesometrial resection (PMMR) in 33.5% of the cases (*n* = 216). Radical hysterectomy was performed in 3% of the cases (*n* = 19). Unspecified types of hysterectomy were the least common, occurring in 2.8% of the cases (*n* = 18) (see Fig. 1).

**Fig. 2:**
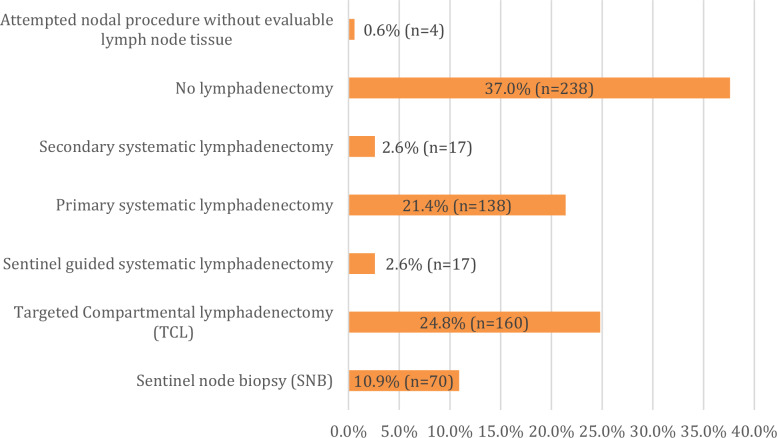
Applied lymph node surgery. Categories are mutually exclusive and refer to patient-level classification. Patients with attempted or documented nodal procedures but without evaluable lymph node tissue on pathological examination were classified separately and considered Nx for TNM nodal staging

Overall, 62.4% (n = 402) of the study cohort underwent lymph node surgery with evaluable lymph node tissue. In an additional four patients (0.6%), a nodal procedure was attempted or documented intraoperatively, but no evaluable lymph node tissue was retrieved or confirmed on pathological examination. In 238 patients (37.0%), no lymph node surgery was performed. The type of lymph node procedures applied is summarized in Fig. 2.

Among patients undergoing lymph node surgery with evaluable lymph node tissue (*n* = 402), 247 (61.4%) received sentinel-based procedures, while 155 (38.6%) underwent systematic lymphadenectomy.

Regarding sentinel-based procedures, the majority of patients (*n* = 160; 64.8%) underwent targeted compartmental lymphadenectomy (TCL). Among patients undergoing systematic lymphadenectomy, most patients (*n* = 138; 89.0%) received primary systematic lymphadenectomy, whereas 17 patients (11.0%) underwent secondary systematic lymphadenectomy. As defined in the Methods section, lymph node procedures were categorized as mutually exclusive at the patient level.

According to TNM criteria, nodal status was classified as Nx in 242 cases (37.6%). This group included 238 patients without lymph node surgery and four patients in whom a nodal procedure was attempted or documented intraoperatively, but no evaluable lymph node tissue was retrieved or confirmed on pathological examination. This distinction between surgical omission, attempted nodal assessment, and pathological nonassessment should be considered when interpreting nodal staging patterns.

**Fig. 3:**
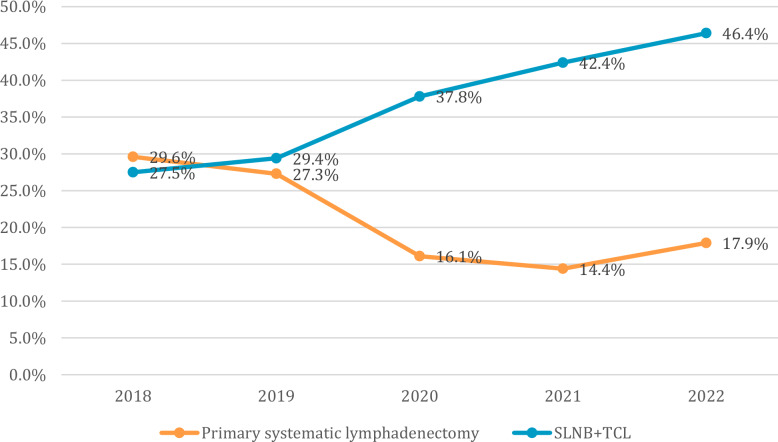
Evolution of primary systematic lymphadenectomy versus SLNB/TCL between 2018 and 2022

The proportion of primary systematic lymphadenectomy decreased from 30.3% in 2018 to 17.9% in 2022 (*p* < 0.05, chi-square test across years). In contrast to systematic lymphadenectomy, the number of less radical procedures such as SLNB and TCL increased significantly between 2018 and 2022 (*p* < 0.05, chi-square test across years) (see Fig. 3).

**Fig. 4:**
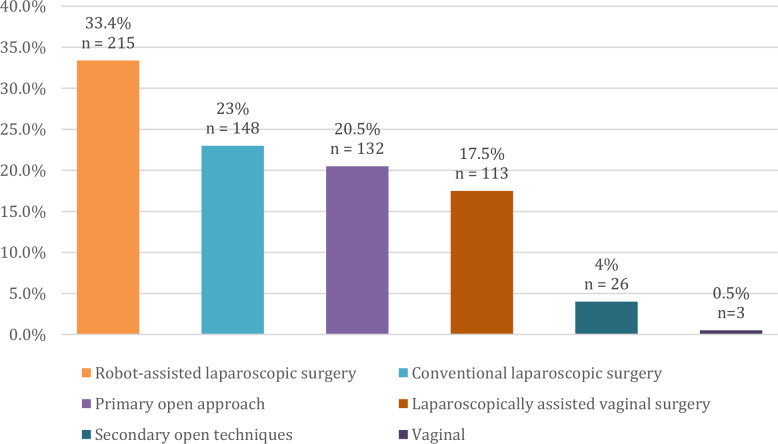
Frequency of access routes for hysterectomies throughout the entire observation period (2018–2022)

It has been demonstrated that throughout the entire observation period, hysterectomies were most frequently performed using robot-assisted laparoscopic techniques (33.4%, *n* = 215), followed by conventional laparoscopic access in 23% of cases (*n* = 148). A primary open approach was chosen in 20.5% of cases (*n* = 132). Vaginal surgery represented the least common category (0.5%, *n* = 3). Secondary open techniques accounted for 4.0% of cases (*n* = 26) (see Fig. 4).

**Fig. 5:**
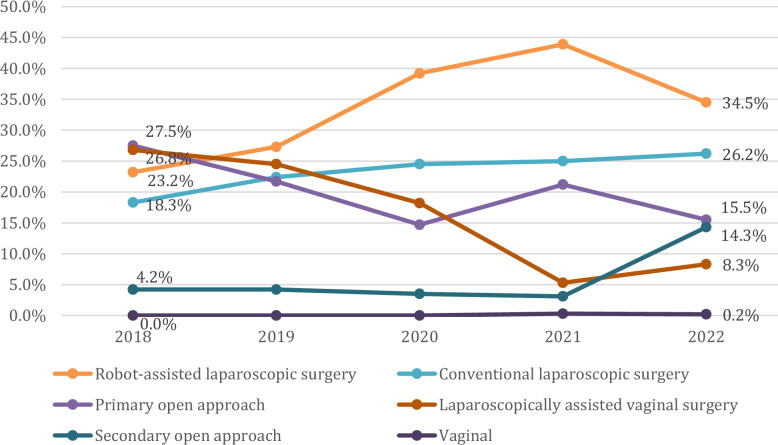
Development of trends of access routes for hysterectomies between 2018 and 2022

Based on a more detailed analysis of the frequency of utilized access routes across the individual years, it is evident that a significant shift in preferred access methods occurred during the observation period. While open access was the most frequently employed route in 2018, it had become one of the less commonly used methods by 2019. There was an increase in the frequency of robot-assisted laparoscopic and conventional laparoscopic procedures over the 5-year period. In contrast to minimally invasive access routes, the number of primarily open approaches and laparoscopically assisted vaginal surgeries decreased between 2018 and 2022 (see Fig. 5).

## Discussion

The demographic data of the current cohort showed a median age of 63 years and a median BMI of 30 kg/m^2^. These findings are consistent with comparable studies, which also report a median age around 63 years and a median BMI of 30 kg/m^2^ [13, 14].

The elevated median BMI suggests a high prevalence of overweight within this population. According to the guidelines on endometrial cancer, obesity is considered an independent risk factor for the development of this carcinoma [15]. Adipose tissue contains high levels of the enzyme aromatase and thus facilitates the conversion of androgens to estradiol, which has a proliferative effect on endometrial cells and consequently increases the risk for endometrial carcinoma. The elevated BMI observed in our cohort is consistent with the known epidemiological association between obesity and endometrial cancer reported in previous studies. However, the present study design does not allow conclusions regarding causality or disease risk.

Morice et al. reported that endometrial carcinoma predominantly occurs in postmenopausal women, with an average age at diagnosis of approximately 60 years. The increased incidence observed in older populations is primarily attributed to hormonal alterations during postmenopause, notably the persistent influence of estrogen in the absence of progesterone [16, 17].

The most frequently performed surgical procedure was simple hysterectomy, followed by PMMR in approximately one-third of cases. Radical hysterectomies were less commonly performed. Similar distributions have been reported in comparable studies [18].

It should be noted that PMMR is a novel surgical approach based on cancer-field concepts, which is currently under evaluation in a multicenter trial (DRKS00016541) and is not yet implemented in clinical guidelines.

Notably, PMMR and targeted compartmental lymphadenectomy are not currently recommended in international guidelines. Their relatively high frequency in this cohort likely reflects center-specific surgical expertise and participation in ongoing research initiatives.

Cancer-field surgery aims at optimal locoregional control by complete resection of the tumor-bearing compartment (“cancer field”) [19].

Because PMMR and TCL were predominantly performed in highly specialized academic centers, center-specific surgical philosophies may have influenced the observed temporal trends, particularly the increase in sentinel-based procedures. Therefore, the generalizability of these findings to nonacademic or lower-volume settings should be interpreted with caution.

The predominance of conservative surgical approaches can be attributed to the fact that endometrial carcinoma is diagnosed at FIGO stage I in approximately 75% of cases, with tumor confinement primarily limited to the local region and minimal spread. Therefore, treatment with a simple hysterectomy is generally considered sufficient for managing early-stage disease, in accordance with current guidelines [15, 20]. Recently published data showed benefits in cancer-specific survival and disease-free survival in patients undergoing radical hysterectomy in clinical stage II endometrial cancer with aggressive histology [21].

Furthermore, there has been a notable increase in the adoption of minimally invasive procedures during the period from 2018 to 2022, concomitant with a decline in open surgical methods. A study by Yoshida et al. described the evolution of hysterectomy techniques in Japan, revealing a progressive shift toward minimally invasive approaches as open procedures became less prevalent [22]. This trend is further supported by findings from Kim et al., who identified technological advancements, such as laparoscopy and robotic-assisted surgery, as key factors facilitating this transition [23]. These minimally invasive techniques are associated with several benefits, including improved patient outcomes, reduced complications and faster recovery times. Additionally, clinicians increasingly favor less invasive options due to patient preferences for reduced pain and shorter convalescence periods. Economic factors also play a role; decreased hospital stays and complication rates contribute to lower healthcare costs. Collectively, these factors have significantly transformed surgical practice patterns in the management of endometrial cancer [24–28].

In this multicenter retrospective cohort, we observed a temporal increase in sentinel-based lymph node procedures and a decline in primary systematic lymphadenectomy between 2018 and 2022. The use of mutually exclusive patient-level classification allowed for a clear comparison of temporal trends in lymph node procedures. The observed trends may also be influenced by changes in patient characteristics over time (e.g., stage distribution, BMI, age), which were not adjusted for in this analysis and may partly explain differences in surgical approaches. Furthermore, the high proportion of Nx cases warrants cautious interpretation of the observed trends. Missing nodal information may reflect omission of lymph node staging in selected low-risk patients, incomplete documentation, or differences in institutional practice patterns, thereby potentially biasing the apparent degree of surgical de-escalation.

Several studies support sentinel-node biopsy in endometrial cancer [24–28]. Matsuo et al. examined trends and outcomes related to SLNB for stage II endometrial cancer. The study population was larger than the cohort in this analysis, and they only analyzed T2 endometrial cancer, in contrast to our cohort, which included all T-stages. The authors described that the utilization of SLNB in stage II endometrial cancer increased significantly over time in the United States. They also observed that, by 2017, the frequency of SLNB alone had surpassed that of SLNB performed with concurrent lymphadenectomy.

Furthermore, their study indicated that incorporating SLNB for nodal assessment was not associated with worsened short-term survival compared to traditional lymphadenectomy [29]. Similarly, the SENTI-ENDO study showed that SLNB in early-stage endometrial cancer is a reliable alternative to complete lymphadenectomy, with high sensitivity and fewer complications [4].

The importance of sentinel lymph node dissection in endometrial carcinoma has only been fully elucidated in recent years through pivotal studies, leading to its gradual integration into both German and international clinical guidelines. The large multicenter FIRES study showed that SLNB has a high detection rate and diagnostic accuracy using indocyanine green, enabling it to safely replace lymphadenectomy for staging [28]. In 2018, German S3 guidelines recommended systematic lymph node dissection for high-risk endometrial tumors—those with higher grade, deeper myometrial infiltration, or other risk factors. For low-risk tumors, full dissection can be avoided to reduce complications, and sentinel lymph node biopsy may be used to assess lymph node status [3]. Furthermore, the SHREC study confirmed that SLNB, with complete sensitivity in detecting pelvic lymph nodes, is reliable in high-risk endometrial cancer and can safely replace a full lymphadenectomy [9]. In 2021, ESGO guidelines recommended systematic lymph node dissection for high-risk endometrial carcinoma—characterized by high tumor grade, deep myometrial infiltration, or other risk factors—to assess metastasis. For low-risk tumors with low grade and no deep infiltration, full dissection can be omitted, and sentinel lymph node biopsy is advised as a less invasive alternative with fewer complications [30]. Compared to the 2018 S3 guidelines, the 2022 update emphasizes a more risk-adapted approach to lymph node management in endometrial carcinoma. While earlier recommendations strongly favored systematic lymphadenectomy for high-risk cases, the new guidelines place greater emphasis on sentinel lymph node biopsy, especially in low-risk patients, to reduce invasiveness and potential complications. Overall, the 2022 guidelines advocate for personalized treatment strategies based on individual risk profiles, with a stronger focus on modern diagnostic techniques [31].

The ESGO recommendations of 2025 continue to emphasize the use of sentinel lymph node biopsy in early-stage disease. Systematic lymphadenectomy is not recommended; only suspicious lymph nodes should be resected as part of the cytoreductive procedure in advanced disease [32].

Several studies have investigated the oncological outcomes of SLNB in patients with high-risk endometrial cancer.

These authors have demonstrated that, in cases of high-risk endometrial cancer, there is no significant difference in clinical outcomes between patients undergoing sentinel lymph node biopsy and those undergoing conventional lymphadenectomy [33, 34].

Overall, there is a trend toward less invasive methods that prioritize patient quality of life; however, long-term data regarding survival outcomes, disease recurrence and quality of life remain under investigation [35, 36].

Future research should focus on these long-term endpoints and include randomized controlled trials to further validate the safety and efficacy of SLNB across diverse molecular risk groups.

In summary, current trends reflect a shift toward minimally invasive procedures designed to reduce treatment-related morbidity without compromising oncological safety or treatment efficacy.

## Limitations

This study has several limitations. First, its retrospective design introduces potential selection bias and confounding. Second, the multicenter nature of the study may reflect center-specific practices and may not be fully representative of broader clinical settings.

No adjusted analyses were performed to account for differences in patient characteristics (e.g., age, BMI, tumor stage) or center-specific effects, which may have influenced the observed temporal trends.

Importantly, a substantial proportion of patients had missing nodal status (Nx, 37.6%), which limits the interpretation of nodal staging patterns and comparisons between surgical strategies. Missing nodal data were likely not random and may reflect differences in surgical indication or patient selection.

Furthermore, although lymph node procedures were categorized as mutually exclusive at the patient level, classification of complex or combined surgical approaches may still be subject to residual misclassification.

In addition, detailed data on sentinel lymph node mapping techniques, including tracer type, detection rates, and laterality, were not consistently available across participating centers.

Molecular classification data were not consistently available across participating centers and therefore could not be incorporated into the present analysis. Given the increasing importance of molecular risk stratification in endometrial cancer, this limits interpretation of surgical decision-making and risk-adapted lymph node staging.

This limits the ability to further assess the quality and consistency of sentinel-based approaches in this cohort.

## Strengths

The strengths of this study include its multicenter design and systematic data collection from six leading German university hospitals, offering a comprehensive view of practice patterns. The use of a shared multicenter database facilitated harmonized data collection across participating centers. However, the retrospective design precluded formal monitoring of data completeness and quality.

Covering 5 years allows for accurate assessment of trends in lymph node evaluation, hysterectomy types, and access routes in endometrial cancer management. These factors enhance the robustness and relevance of the findings.

## Conclusion

In this multicenter retrospective cohort, we observed a temporal increase in sentinel-based lymph node procedures and a decrease in systematic lymphadenectomy between 2018 and 2022.

These findings describe evolving surgical practice patterns; however, causal interpretation is limited by missing data, lack of adjusted analyses, and potential center-specific effects.

## Data Availability

The dataset generated and analyzed during the current study is not publicly available due to data protection regulations and institutional restrictions. The data may be made available from the corresponding author upon reasonable request, subject to approval by the participating institutions and compliance with applicable data protection requirements.
